# Wherefore Art Thou, Homeo(stasis)? Functional Diversity in Homeostatic Synaptic Plasticity

**DOI:** 10.1155/2012/718203

**Published:** 2012-05-17

**Authors:** Bridget N. Queenan, Kea Joo Lee, Daniel T. S. Pak

**Affiliations:** ^1^Department of Pharmacology and Physiology, Georgetown University Medical Center, 3900 Reservoir Road, NW, Washington, DC 20057, USA; ^2^Interdisciplinary Program in Neuroscience, Georgetown University Medical Center, 3900 Reservoir Road, NW, Washington, DC 20057, USA

## Abstract

Homeostatic plasticity has emerged as a fundamental regulatory principle that strives to maintain neuronal activity within optimal ranges by altering diverse aspects of neuronal function. Adaptation to network activity is often viewed as an essential negative feedback restraint that prevents runaway excitation or inhibition. However, the precise importance of these homeostatic functions is often theoretical rather than empirically derived. Moreover, a remarkable multiplicity of homeostatic adaptations has been observed. To clarify these issues, it may prove useful to ask: why do homeostatic mechanisms exist, what advantages do these adaptive responses confer on a given cell population, and why are there so many seemingly divergent effects? Here, we approach these questions by applying the principles of control theory to homeostatic synaptic plasticity of mammalian neurons and suggest that the varied responses observed may represent distinct functional classes of control mechanisms directed toward disparate physiological goals.

## 1. To Take Arms against a Sea of Troubles, and by Opposing End Them: Homeostatic Self-Regulation in Neurons

The concept of homeostasis has become a central tenet of physiology in the 80 years since its formal articulation [1]. Homeostatic regulation dynamically maintains the relatively fixed *milieu intérieur* which the French physiologist Claude Bernard defined as “the requirement for a free and independent life” [2]. However, the notion of neuronal homeostasis is a relatively new variation on this theme. In the past two decades, neurons and neuronal networks have been observed to self-regulate their output in a variety of in vitro and in vivo contexts. Despite (or because of) the explosion of research in recent years, homeostatic adaptation of neuronal synapses (known collectively as homeostatic synaptic plasticity or HSP) resists easy packaging into an overarching model, but instead seems splintered into a complex array of different factors and multiple mechanisms [3–5]. Here, we critically survey the literature and attempt to synthesize these varied observations into a more coherent picture by asking what purpose homeostatic adaptations serve. To limit the overwhelming number of questions raised by these issues, we restrict our focus to the best-characterized form of adaptation, the homeostatic responses occurring at excitatory synapses of the mammalian central nervous system (CNS). Other recent reviews have extensively covered other aspects such as intrinsic excitability [4, 6, 7], excitation-inhibition balance [4, 5], and the catalog of various molecules implicated in homeostatic adaptation [3, 8], and we have not attempted to provide a comprehensive review of these topics. To begin, we will apply the conceptual lens of control theory, which may provide a helpful framework in attempting to develop unifying organizational principles. We then attempt to explicate the variability of homeostatic responses as distinct methods of accomplishing multiple biological functions or goals in different cell types and circuits.

## 2. HSP: Lessons from Engineering

 A critical question that often goes unanswered, or at least is left implicit, is that of the physiological importance of HSP. Theoretical network models suggest that HSP is a key ingredient for optimal information processing and stability. A popular view is that HSP is a requisite negative feedback “yin” to the “yang” of positive feedback-based associative, or Hebbian, plasticity mechanisms such as long-term potentiation (LTP) and long-term depression (LTD) [3, 5, 7, 9]. However, we can envision several scenarios in which different classes of homeostatic regulation may plausibly play biologically important roles, as discussed in the sections below. 

**Figure 1:**
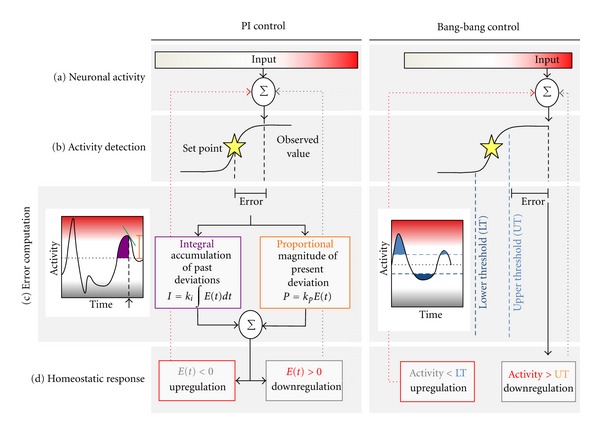
Closed-loop control in homeostatic regulation. In closed-loop control systems, observed activity values (a) are compared to a desired set point (yellow star) (b) and deviations are registered as errors (c). The homeostatic response program is calculated and initiated in response to the error signal (d). Many control strategies are possible, including proportional-integral (PI) control (left) and bang-bang control (right). *PI control*: PI controllers compute a compensatory response as a function of the properties of the error, namely, the proportional (orange, magnitude at *t* = 0 indicated with arrow) and integral (purple, cumulative error over time) components of the deviation. A variation of this regulation, the proportional-integral-derivative (PID) controller, also incorporates a derivative component that detects the rate of change of the deviation (green bar in activity trace, *D* = *k*_*D*_ *dE*(*t*)/*dt*). The initiated response is therefore tailored to the immediate degree of deviation from the set point (proportional), the cumulative magnitude of the deviation (integral), and the rate of change of the deviation (derivative). *Bang-bang control*: Bang-bang control consists of set compensatory responses which are initiated once a threshold is crossed (blue lines) and halted once the activity value returns to the acceptable range of values.

In elucidating these functions, we propose to use the context of control theory [10–12]. Although such engineering models have been conceptually applied to homeostatic behavior of physiological systems [13–15] including neural ones [16, 17], in this paper we will systematically examine the literature via the lens of closed-loop control to explicate the abundant body of existing data. In a closed-loop control system, a *sensor* monitors system output and feeds the information back to a *controller* which adjusts one or more control parameters to maintain output at a desired level. This mode of regulation allows the controller to compensate dynamically for changes to the system by “looping back” to modulate control and contrasts with *open-loop* systems that lack such feedback mechanisms. Closed-loop regulation of neuronal activity can be broken down into the following parts: (1) detection of a specific output measure of activity (Figure 1(a)), (2) comparison to a set point representing “optimal” activity (Figure 1(b)), (3) calculation of “error,” or the difference between detected and optimal levels, and computation of an appropriate homeostatic response program tailored to the error (Figure 1(c)), and (4) implementation of the compensatory homeostatic response (Figure 1(d)). The mechanisms of homeostatic control will likely depend critically and differentially on the physiological conditions that trigger different types of HSP in specific situations and contexts.

### 2.1. What Do We Mean by Activity?

Publications in the field of homeostatic synaptic plasticity invariably begin with the statement that neurons, faced with chronic changes in activity, alter their properties to return their output to normal levels. However, it is unclear what exactly is meant by terms such as “activity,” “output,” and “normal.” Homeostatic regulation is predicated on the notion that biological systems have ideal set points for various parameters and that these set points are dynamically maintained to allow for continued function despite constantly fluctuating external environments. This notion seems intuitive for systems that require robust and relatively stable output, such as the neuromuscular junction [16, 18]. However, it is less obvious how homeostatic plasticity may be implemented in complex, highly plastic, and information-encoding environments such as the mammalian CNS. 

It is immediately apparent that neuronal “activity” could be defined in a multitude of ways within a single (excitatory) cell, including currents or membrane potential fluctuations in postsynaptic, dendritic, or somatic membranes; calcium flux in dendritic spines, dendrites, or soma; action potential generation (i.e., firing rate) at the axon hillock; vesicular accumulation and release at presynaptic terminals; neurotransmitter concentration within or near the synaptic cleft, just to name a few. Each of these locations may have distinct set points and sensors for determining activity status, governing independent or concerted forms of plasticity that coexist within the neuron and that may be called upon during different functional requirements.

### 2.2. How Are Errors Detected?

Neurons undergo fluctuations in synaptic input and action potential firing on a scale of seconds to minutes that are required for normal neuronal function. However, at what point has an activity regime switched over from “acute” to “chronic” or from “normal” to “excessive”? Alternatively, is there continual HSP adjustment occurring in proportion to activity levels? This measure will clearly depend on the cell type in question and its preferred firing rate/pattern, but are there general mechanisms that detect aberrant neuronal activity?

In man-made process control, several homeostatic strategies are widely applied depending on the particular requirements. Bang-bang control (Figure 1, right) is a relatively simple control strategy used by thermostats to homeostatically regulate temperature: compensatory responses turn on when a threshold is exceeded (or after a certain delay) and turn off when the set point is achieved. Another common approach is proportional-integral control (Figure 1, left), which initiates feedback tailored to the properties of the detected error. The proportional component reflects the current degree of deviation from the ideal, while the integral component senses accumulation of errors over time and exerts greater feedback as the sum of these errors increases. Thus, the compensatory response is a function of both the magnitude and persistence of the deviation from the set point. Computational studies on homeostatic neuronal activity regulation have demonstrated that directly linking an activity measure (somatic Ca^2+^ levels) to the conductances of ion channels confers integral control over the channel without explicit integration [19, 20]. It should be stressed that there is an infinite number of possible control methods and that these examples given are not necessarily the ones used in biology, but may serve to provide valuable conceptual guidance when approaching distinct types of homeostatic regulation.

### 2.3. What Are the Compensatory Responses?

As we have outlined, the set point for neuronal “activity” could potentially consist of multiple parameters including synaptic currents, calcium levels, action potential firing rate, presynaptic vesicle number, or neurotransmitter concentration. Theoretically, these same parameters could be altered to homeostatically adjust neuronal “output,” but this does not necessarily have to be the case. In order to regain appropriate activity state, neurons and networks can alter basically all of their components: passive and active membrane properties [6, 7, 21, 22], densities and conductances of ion channel subtypes [23, 24], efficacy and locations of inhibitory and excitatory connections [25–27], modulatory neurotransmitter (dopaminergic/serotonergic/acetylcholinergic) tone, and so forth. Indeed, network simulations suggest that a large number of combinatorial parametric modifications can yield equivalent neuronal firing pattern corrections [28]. These diverse participants in homeostatic adaptation no doubt contribute to the large number of mechanisms being uncovered, which may act coordinately or as multilayered back-up systems in case of failure or overload in primary ones. As we have stated earlier, here we will focus on a small sector of this parametric space, the changes occurring at excitatory synapses.

## 3. Whys and Wherefores of Excitatory HSP: Functional Classification

Activity at excitatory synapses consists primarily of excitatory postsynaptic currents (EPSCs) mediated by the AMPA subtype of fast glutamate receptors (AMPARs), and in the CNS occurs predominantly at small, motile protrusions called dendritic spines. Characteristics of AMPAR-mediated miniature EPSCs, the postsynaptic responses to release of a single presynaptic vesicle of glutamate, are widely used to infer information about synaptic properties. Increases in mEPSC amplitude are consistent with higher density/conductance of postsynaptic receptors at individual synapses [23]. Elevated mEPSC frequency is usually interpreted as increases in either the presynaptic release probability at existing sites (increases in the vesicular pool or vesicular turnover rate) [29] or in the number of functional synaptic sites (more dendritic spines or new synapses onto already established spines) [30]. Accordingly, decreases in mEPSC amplitude and frequency, observed in various overactivity paradigms, are interpreted as decreases in postsynaptic and presynaptic properties, respectively. However, caution is needed in attributing mEPSC alterations to exclusively pre/postsynaptic changes. For instance, mEPSC frequency and amplitude are not independent; practically speaking, once synapses become very small, current amplitudes from them fall below the threshold of detection and this leads to a decrease in the measured frequency. Additionally, unsilencing of so-called “silent” synapses that previously lacked AMPARs [30] is a postsynaptic effect which manifests as a change in mEPSC frequency. Furthermore, there is evidence that pre- and postsynaptic development is coordinated [31, 32]. With these caveats in mind, it is still controversial whether changes in mEPSC amplitude or frequency are the predominant HSP response and which AMPAR subunits are the main players subject to regulation (e.g., [23, 24, 33–36]; see Table 1). As we shall see, careful consideration of experimental variables and biological functions may shed light on these and other controversies. In the following sections, we will explore several possible neuronal contexts for HSP, using control theory to dissociate the components involved and delineate the different functional classes (summarized in Table 2). In particular, we will examine various inactivity paradigms with respect to three variables: scope, synaptic locus, and degree. The scope of the inactivity may be either network-wide (Section 4), cell autonomous (Section 5), or synapse specific (Section 6). Within each section, we will examine divergent findings by grouping inactivity paradigms by synaptic locus (pre- or postsynaptic) and degree (reduced or abolished activity). The focus on inactivity regimes reflects the preponderance of studies using these experimental paradigms, although in some cases we will delve into the consequences of overactivity. When appropriate, we will distinguish between developing and established networks, as the developmental state of the network has consequences for functional regulation. 

## 4. Network-Wide Inactivity

The most commonly used inactivity paradigms induce network-wide changes in activity state via bath application of drugs. In the following sections, we group the network-wide inactivity paradigms into functional classes (see Table 2) according to the locus (pre- or postsynaptic) and severity (reduced or abolished) of the inactivity

### 4.1. Reduced Presynaptic Input: Scaling and Synaptic Calibration

A seminal observation in the field was that simply seeding cultured hippocampal neurons at different densities caused reciprocal regulation of synaptic strength, with higher densities yielding weaker synapses and lower densities resulting in stronger synapses [59]. A similar result is found when cells are plated onto larger surface areas, even when the cell density remains constant: large networks have more heavily interconnected neurons with globally weaker excitatory connections and stronger inhibitory connections; conversely, smaller networks have less synaptic innervation but proportionally stronger constituent excitatory connections and weaker inhibitory ones [26]. The network size and the degree of innervation therefore control the range of synaptic strengths that are considered to be acceptable in the first place, presumably functioning as a guard to maintain stability during development of networks.

A global form of synaptic adaptation can also be induced pharmacologically, via application of the voltage-gated sodium channel blocker, tetrodotoxin (TTX), which prevents the firing of action potentials (APs) and thus greatly reduces the frequency of presynaptic vesicular release. Much of the early work on describing the effects of HSP on excitatory synapses was performed in young neurons, during the period of robust synaptogenesis, which occurs in vivo at 2 weeks postnatal [60] and at day in vitro (DIV) 10–14 [61]. In dissociated primary cultures of young cortical [38, 40, 41] or hippocampal [41, 44] neurons, TTX has been observed to cause a global increase in AMPAR-mEPSC amplitudes but no change in frequency, suggesting increased postsynaptic strength but not synapse number or release kinetics. Such a phenomenon has led to the prevailing notion of “synaptic scaling” [38], a neuron-wide, multiplicative change in synaptic strength at all synapses. The finding that networks calibrate the strength of their synaptic connections raises the possibility that TTX blockade during development, by reducing the frequency of synaptic inputs, “tricks” neurons into believing they are part of a less dense neuronal network. The resulting scaling in synaptic strength could therefore be considered part of a developmental synaptic calibration machinery.

How does the neuron sense its total endowment of synaptic innervation? In the case of global homeostasis, one possible activity sensor is postsynaptic firing rate, as compensatory neuron-wide HSP can be initiated by local application of TTX to neuronal cell bodies but not to portions of the dendritic tree [40]. However, sustained postsynaptic depolarization is sufficient to induce downregulation of synaptic strength independent of action potential firing [62]. This conclusion has been supported by a recent study demonstrating that chronic optogenetic overactivation of individual CA1 neurons in hippocampal organotypic slices induces cell autonomous homeostatic downregulation of postsynaptic strength [63]. This leads to the question of what is actually being measured and how this translates into an index of over- or underactivity. Somatic calcium levels appear to be an important activity sensor in this process [8, 40, 63], and L-type voltage-gated Ca^2+^ channels have been implicated as the mode of calcium entry [24, 39, 46, 63]. Downstream calcium-dependent second messengers such as calmodulin [64] or various enzymes (e.g., adenylyl cyclase [39]) could represent biochemical readouts of these calcium transients. Interesting examples of the latter category are *α*- and *β*-CaMKII, prominent Ca^2+^/calmodulin-dependent postsynaptic kinases that are reciprocally downregulated and upregulated, respectively, during prolonged inactivity [51], and are associated with L-type voltage-gated Ca^2+^ channels [65].

The related family member CaMKIV also appears to be an important potential sensor, as its function is required for homeostatic downregulation in response to optogenetic hyperstimulation [63], while decreased nuclear CaMKIV activation mimics and occludes adaptation to neuronal inactivity [40]. Because gene transcription [40, 44] or protein translation [35, 49, 50, 66] is required for some forms of HSP, a potential integrative mechanism for registering and integrating errors in activity state could be based on the accumulation of activity-dependent mRNAs or proteins. Such a system may involve activity-inducible inhibitory factors such as the immediate early gene Arc [52, 67], inactivity-induced stimulatory factors, or both for optimum bidirectionality. For example, polo-like kinase Plk2 transcription is tightly regulated by neuronal activity and, upon induction, downregulates excitatory synapses and dendritic spines [68–72]. Thus, the amount or balance of these factors could establish the length of time and/or extent of deviation from the desired set point.

### 4.2. Reduced versus Abolished Postsynaptic Activity: Global versus Local HSP

Various activity sensors and homeostatic mechanisms can be pharmacologically dissected using antagonists of specific ion channels. TTX initiates slow compensatory responses in AMPAR mEPSC amplitude on the scale of 12–48 hrs in developing hippocampal neurons (e.g., [38, 45]; see Table 1). The time course of adaptation can be rapidly accelerated to 4 hours or less by blockade of glutamatergic synaptic transmission with antagonists of AMPARs [49] or concurrent application of TTX with NMDAR antagonist APV [35, 49]. Interestingly, NMDAR blockade alone did not appear to induce a homeostatic AMPAR response in at longer time points in developing cortical neurons [38, 39, 46].

Not only the timecourse but the compensatory response varies between inactivity paradigms. AMPAR blockade alone induces an increase in both mEPSC frequency and amplitude (e.g., [24, 46, 49]; see Table 1 for others), suggesting concerted pre- and postsynaptic adaptations to inactivity. TTX by itself generally induces an increase only in mEPSC amplitude (e.g., [38, 46, 49]; see Table 1 for others), suggesting a predominantly postsynaptic response. Furthermore, treatment of mature hippocampal neurons with TTX together with the selective AMPAR blocker NBQX has been found to be actually *subtractive* [46, 49]. TTX appeared to block the NBQX-induced changes in frequency [46, 49], supporting the notion that the coordination of presynaptic function with postsynaptic status requires ongoing AP firing [31, 73, 74], possibly due to the state-dependent interaction of presynaptic terminals with inactivity-released dendritic BDNF [49].

A drawback to the use of bath application of drugs is that these manipulations are not particularly “clean,” in that TTX and NBQX will both reduce synaptic input and action potential firing either directly or indirectly. Nevertheless, the combined pharmacological manipulations reveal that the inactivity induced with TTX is not equal to the inactivity induced with glutamatergic receptor blockade, suggesting that somatic and synaptic activity may be differentially regulated. Indeed local TTX blockade of somatic activity is capable of inducing neuron-wide scaling [40], while local TTX blockade of dendritic activity does not induce upregulation. To our knowledge, neuron-wide scaling in response to the over- or underactivity of a subpopulation of synapses (as might result from input-specific Hebbian modifications) has not been reported.

What is the biological significance of these two mechanisms? Decreased AP firing (due to TTX treatment) can be interpreted by a receptive neuron as a deficiency of postsynaptic function, thus resulting in a slow upregulation of AMPAR synaptic content. The silencing of AMPAR transmission (due to NBQX treatment) could therefore represent the most extreme end of this postsynaptic deficit spectrum. The magnitude of input “error” resulting from complete AMPAR blockade would be considerably larger than that from TTX blockade, leading to a correspondingly faster rate of the response. The existence of a minimum postsynaptic activity threshold (e.g., calcium) could explain why APV and TTX together are able to induce rapid responses, while neither do so alone.

However, it seems that the two responses to TTX- and NBQX-induced inactivity have different underlying compensatory mechanisms and likely achieve separate physiological goals. The rapid HSP induced by glutamatergic receptor blockade appears to specifically involve enhanced GluA1 synthesis and synaptic incorporation of Ca^2+^-permeable GluA2-*lacking* AMPARs [24, 33, 35, 36], whereas the slow HSP induced by TTX generally increases both GluA1 and GluA2 subunits [23, 34, 35] and in fact selectively requires the GluA2 C-terminal tail [77]. Since the homeostatic responses differ in these two activity paradigms, it is possible that distinct mechanisms are recruited in the fast and slow forms of HSP. We note that complete cessation of AMPAR- or NMDAR-mediated transmission is not a physiological response under normal circumstances. Perhaps such inactivity occurs when existing synapses become damaged, defective, or otherwise nonfunctional, and the rapid response to these manipulations could therefore represent emergency synaptic “repair” mechanisms. A bang-bang control strategy would be ideal for implementing such pathways. Glutamatergic receptor blockade has been shown to induce dendritic translation of retinoic acid [46, 50] and the multifunctional neurotrophin BDNF [49], both of which have been shown to play roles in HSP. These dendritically synthesized proteins potentially function in a form of bang-bang control of local synaptic strength, in which dendritic protein synthesis is turned on once local Ca^2+^ levels have dropped below a certain threshold and is turned off once newly inserted Ca^2+^ permeable AMPARs allow for sufficient Ca^2+^-influx. In contrast, somatic Ca2+ levels may be monitored continually on slower timescales by a somatically deployed PI control mechanism.

### 4.3. Abolished Presynaptic Activity?

 Existing global inactivity paradigms reduce or block postsynaptic activity (Section 4.2), and reduce presynaptic activity (Section 4.1). Global cessation of presynaptic input has not been reported, but could potentially be achieved by infecting cultured neurons at sufficiently high titer of viruses expressing tetanus toxin to inactivate all presynaptic vesicular release in the culture. This manipulation might be useful to dissect the effects of presynaptic activity versus presynaptic neurotrophic support.

## 5. Cell Autonomous Inactivity: Synaptic Competition versus HSP

In contrast to the global amplitude effects observed in developing networks treated with TTX, a different outcome is observed when the excitability of a single neuron is reduced by transfection of hyperpolarizing potassium channel Kir2.1 [45]. Expression of the channel in cultured hippocampal neurons prior to extensive synaptogenesis did not induce homeostatic upregulation, instead causing a reduction in the number of functional excitatory synapses onto the transfected cell and smaller presynaptic boutons, with no change in mEPSC amplitude. This non-homeostatic effect appeared to be due to developmental competition among neurons for inputs, as this imbalance in synapse formation was eliminated if all cells were inhibited with TTX. Interestingly, expression of the Kir2.1 channel *after* the bulk of synapse formation initiated a homeostatic upregulation of presynaptic function (increased AMPAR-mEPSC frequency due to a larger vesicle pool and presynaptic release probability), with no change in synapse number or mEPSC amplitude. The presynaptic homeostatic adjustment appears to fully compensate for the initial reduction in postsynaptic activity, as the firing rate of Kir2.1-transfected cells eventually returns to control values. In this scenario, the functional deficit induced by Kir2.1 can be viewed as decreased postsynaptic efficacy with normal presynaptic function. Why then does the inhibited neuron not initiate a global synaptic scaling of AMPAR-mEPSC amplitudes, as observed with TTX? It is possible that the effect of Kir2.1 is less severe than TTX and does not reduce somatic calcium sufficiently to induce a scaling response. Another possibility is that the severe decrease in presynaptic release due to TTX treatment results in compensatory boosting of the properly functioning postsynaptic side, whereas the postsynaptic impairment from Kir2.1 hyperpolarization is combated via compensatory upregulation of the unperturbed presynaptic apparatus.

## 6. Synapse-Specific Inactivity

### 6.1. Reduced Presynaptic Input: Synapse-Specific HSP

A prediction from synaptic scaling is that activity changes at any given synapse do not initiate global homeostatic compensation, as the neuron is somatically monitoring the sum of all synaptic activity and coordinating any necessary homeostatic adaptation among all ~10,000 synapses of a typical neuron. This prediction is borne out by several studies that show that local synaptic inactivation does not cause global scaling [40, 75, 76]. However, modulation of single synapses does yield input-specific effects. The activity of individual presynaptic terminals can be decreased due to presynaptic neuronal hyperpolarization via sparse transfection with the rectifying potassium channel Kir2.1 [47, 52]. In young hippocampal neurons, the rare postsynaptic targets of the selectively depressed presynaptic neuron's terminals homeostatically upregulated their AMPAR content and strength, though neighboring synapses did not, in a process involving GluA2-lacking receptors and Arc [47, 52].

What might be the functional importance of this synapse-specific homeostatic control? We suggest that scaling and synapse-specific HSP are dual mechanisms that operate in tandem in developing neurons to establish proper network and synaptic functionality. During synapse formation, one may imagine that it would be useful to employ a program of synaptic quality control during the construction of an appropriately functioning synaptic tree. Scaling may be responsible for globally establishing and maintaining an appropriate set point (or rather a set range) for synaptic strengths, based on the total innervation pattern and firing rate of the cell. Meanwhile, synapse-specific HSP may represent the means of adjusting individual synaptic strengths to values within the globally established range that are most appropriate based on the activity of the corresponding pre/postsynaptic terminal and on that of neighboring synapses. The AMPAR content of excitatory synapses appears to consist of both “stable” and “labile” populations [78]. The labile population may be a more dynamic, heterogeneous set of receptors that can be mobilized by Hebbian or synapse-specific homeostatic plasticity, whereas the size of the core stable AMPAR population may be established during development in a relatively standardized way throughout the dendritic tree.

### 6.2. Abolished Presynaptic Input

Interestingly, completely abolishing presynaptic vesicular release does not merely exaggerate the response seen with diminished presynaptic release. Instead, seemingly opposite effects are observed if presynaptic vesicular release is abolished (using tetanus toxin) rather than diminished (using presynaptic Kir2.1). If a similar presynaptic manipulation is performed as in Section 6.1, using instead tetanus toxin to completely inactivate presynaptic terminals, no change in AMPAR-mediated currents is observed [79], only a specific reduction in GluA1 (and not GluA2/3) AMPAR subunits [76], likely involving increased diffusional exchange of this AMPAR subunit [75]. As in the postsynaptic scenario discussed in Section 5, input blockade may not represent merely a more extreme portion of the signaling spectrum. The absence of any activity emanating from the presynaptic terminal may be a qualitatively different activity signal than a simply a decrease in presynaptic release. Indeed, abolished (not diminished) presynaptic activity may indicate a nonfunctional presynaptic terminal, in which case postsynaptic homeostatic compensation would be futile. The loss of GluA1 in this context would therefore not represent a homeostatic response, but a lack of activity-dependent GluA1 trapping [75]. It is conceivable that in this situation mechanisms are initiated to upregulate or “repair” presynaptic activity but are obscured by the inability of the system to overcome the inhibition of the tetanus toxin. 

### 6.3. Reduced Postsynaptic Responsiveness

Although local dendritic application of TTX alone did not cause homeostatic responses [40], dendritic application of TTX with the NMDAR antagonist APV induced robust upregulation of surface AMPAR levels in the deprived area [35]. Taken together, these findings suggest that glutamate receptor activity serves as a local signal regulating the strength of individual synapses in an autonomous fashion.

Analogous to the role of somatic calcium in global responses, calcium entry into spines is also likely to play an important role in local synapse-specific regulation. Indeed, the response to AMPA receptor blockade has frequently been detected as the selective insertion of GluR2-lacking AMPA receptors which are Ca^2+^ permeable [24, 33, 35, 36]. These findings suggest that the local synapse-specific responses may be an attempt to restore local Ca^2+^ levels. Local synaptic activity has been heavily implicated in the regulation of dendritic protein synthesis [35, 80]. In fact, miniature synaptic currents have been shown to negatively constrain dendritic protein synthesis, making it possible that the default state of the neuron is to produce proteins for synaptic integration. Postsynaptic activity (in the presence of a functioning presynaptic terminal) may therefore negatively constrain a default program of local homeostatic “upregulation.”

### 6.4. Other Activity Paradigms

 While global hyperactivity paradigms have been shown to induce global decreases in mEPSC amplitude and/or frequency [23, 38, 51, 70], to date, no experiments have examined the effect of synapse-specific overactivation. Chronically increasing presynaptic activity at a single synapse could be accomplished with sustained optogenetic activation of a channelrhodopsin-expressing presynaptic neuron. Homeostatic adaptation to increased activity of a single postsynaptic site has also not yet been reported but may be possible with chronic local uncaging of glutamatergic agonists.

## 7. Nonuniform HSP of Mature Neurons

An appealing theoretical aspect of global multiplicative synaptic scaling is the preservation of the pattern of relative differences in synaptic weights established by Hebbian forms of synaptic plasticity that is postulated to encode information [9]. However, while uniform synaptic scaling has been reproducibly observed in young neurons under appropriate conditions, older neurons (here defined as those beyond the period of bulk synaptogenesis, for example, >DIV21 or in the adult animal) from a variety of preparations do not show scaling, even with global activity manipulations [27, 53, 56, 57]. The occurrence of multiplicative scaling only during the period of peak synaptogenesis (and not in older neurons) suggests that this mechanism may actually be more relevant to synapse formation rather than information processing per se.

Instead, TTX applied to older neurons elicits nonmultiplicative increases in mEPSC amplitudes [56], as well as elevated mEPSC frequency (e.g., [41, 44, 45, 53, 56]; see Table 1 for others). A perplexing question that then arises is that if synapse strength is affected in a nonuniform way, how can homeostatic adjustments coexist with Hebbian information encoding? One proposal for allowing the coexistence of Hebbian and homeostatic mechanisms is if the former is implemented by dynamically moving the set point of the latter [8, 17], in much the same way that a thermostat can be turned up or down, but still remains under feedback control. However, this mechanism does not explain the nonmultiplicative HSP in older neurons. The basis of this HSP in mature neurons remains unknown, but by definition a nonmultiplicative process implies that certain synapses are affected differentially, and in mature neurons HSP has indeed been shown to influence larger synapses disproportionately [24]. The implication of these results is that, in older neurons, some synapses retain higher capacity to generate strong homeostatic responses, while others may become relatively insensitive to chronic changes in activity. We note that the latter population would be ideally suited to durable and persistent information encoding. We speculate that this hypothetical division of plasticity labor would nicely allow homeostatic adjustment without interference with Hebbian plasticity, but such a mechanism remains to be identified and described.

Consistent with the notion that older neurons have populations of synapses that may be resistant to homeostatic adjustment, blocking presynaptic neurotransmitter release at single synapses with tetanus toxin transfection in mature hippocampal neurons did not cause changes in AMPAR-mediated currents at contacting postsynaptic sites but did cause changes in NMDAR subunit composition in an interesting form of metaplasticity or the “plasticity of plasticity” [79]. In older neurons, metaplasticity may provide an attractive alternative (or additional) strategy for restraining the capacity of Hebbian plasticity without interfering with synaptic weighting [7]. Alternatively, changes in presynaptic release probability may allow for homeostatic adjustments without altering postsynaptic information encoding. Indeed, in the intact adult hippocampus, CA1 synapses do not show mEPSC amplitude changes in response to TTX but only increased frequency [56].

In vivo, network stability may also arise as a consequence of the specific arrangement of connectivity and not merely the individual synaptic strengths. For instance, chronic inactivity in mature organotypic hippocampal slices induced upregulation of synaptic efficacy in a manner which reflected the underlying computations of the network. Within the hippocampal trisynaptic circuit, CA3 “throughput” synapses were upregulated in response to inactivity, while recurrent synapses were downregulated [54]. It is therefore possible that, in functional circuits, certain synaptic interfaces are a designated homeostatic locus. Similar synapse-specific adaptations have been detected in the visual system, and interestingly the locus of the homeostatic adaptation appeared to change with development. Visual deprivation induced selective homeostatic adaptation in layer II/III neurons in adult visual cortex, while inducing selective layer IV adaptation in developing neurons [57]. These results suggest not only that multiple HSP mechanisms exist in vivo [27] but also that specific cell types may differentially mediate HSP and that the computations of the network at different developmental time points can alter the locus of homeostatic adaptation.

## 8. Culture Clash: Experimental Preparations

Unlike LTP of hippocampal CA1 synapses, the most well-studied form of plasticity, no standard preparation exists for studies of HSP, leading to experimental variability as noted previously [5]. The problem is particularly acute for cultured cortical or hippocampal neurons, popular but notoriously variable systems for the in vitro study of HSP. Technical aspects of the culture procedures (media preparation, growth substrate, time of culture, age of animals used, culture density or size, etc.) can all influence basal culture properties including synaptic connectivity and strength [26]. The same treatment or combination of treatments can produce different effects in different labs even in what appears to be the same preparation (Table 1).

It should therefore be pointed out that dissociated cultures are not homogenous pools of interchangeable neurons, but are instead highly heterogeneous populations consisting of multiple neuronal types (pyramidal neurons, interneuron subtypes, granule cells, etc.) which vary in proportion depending on the preparation. Rarely do studies attempt to distinguish which cell types are analyzed. Even the balance of glial cells versus neurons can affect synaptic properties and HSP responses [40], since astrocyte- and glial-derived factors regulate scaling of synaptic activity [81, 82]. We therefore emphasize the importance of such variables with the idea that these differences are not simply technical inconveniences but are actually meaningful and can inform our ideas about the functions being supplied under particular circumstances.

## 9. Conclusions and Perspectives

 A great deal of progress has been made in identifying HSP mechanisms and the molecules involved. However, a more careful consideration of the experimental variables of network size, age, and cell type is necessary to clearly parse out the rich and fascinating diversity of homeostatic neuronal adaptations. In developing neurons, the primary goal may be to generate synapses and networks with fidelity and stability, involving neuron-wide regulation of synaptic strength and number. In mature neurons, HSP may be restricted to certain subsets of synapses or cells in an effort to more efficiently respect information encoded in synaptic weights. Thus, framing HSP in biological functions will help understand what goals are sought and hence what underlying mechanisms need to be recruited. Instead of referring by HSP as a monolithic entity, several independent subclasses will likely need to be recognized that operate in different ways. But HSP, by any other name, would be as exciting and interesting an avenue for continued research in the years to come.

## Figures and Tables

**Table 1:** Homeostatic synaptic adaptations to chronic inactivity. An overview of select references which have investigated the neuronal response to chronic inactivity via functional analyses of AMPA receptor-mediated excitatory synaptic transmission. References are arranged by cell type (column 1) and inactivity paradigm (column 2). Within each paradigm, studies are listed in ascending age order (column 3). ↑,↓ = significant change in mEPSC amplitude or frequency. — = no change in parameter. N/A = parameter was not reported. *mEPSC frequency was not directly measured.

Cell type	Inactivity paradigm	Days in vitro (DIV) or postnatal day (P)	Amp.	Freq.	Reference
In vitro (dissociated culture)					

Spinal cord	CNQX + APV	DIV 10	↑	—	[23]

Cortex (Ctx)	CNQX + APV	DIV 21	↑	↑	[37]

Ctx	APV	DIV 7–9	—	—	[38]

Ctx	CNQX	DIV 7–9	↑	—	[38]
		DIV 14–17	↑	↑	[39]

Ctx	TTX	DIV 7–9	↑	—	[38]
		DIV 7–10	↑	—	[40]
		DIV <10	↑	—	[41]
		DIV 11–13	↑	—	[42]
		DIV 14	↑	—	[43]
		DIV >18	↑	↑	[41]

Hippocampus (Hpc)	TTX	DIV 7	↑	—	[44]
		DIV 10	↑	—	[41]
		DIV 14	↑	↑	[45]
		DIV 14	↑	↑	[44]
		DIV 14	↑	—	[35]
		DIV 14	↑	—	[46]
		DIV 14	↑	N/A	[47]
		DIV 18	↑	↑	[41]
		DIV 21-22	↑	—	[48]
		DIV 21	N/A	“↑” *	[29]
		DIV 27–40	↑	—	[49]

Hpc	TTX + APV	DIV 14	↑	—	[35]
		DIV 14-15	↑	—	[50], [46]

Hpc	TTX + CNQX	DIV 14	↑	—	[46]

Hpc	TTX + NBQX	DIV 27–40	↑	—	[49]

Hpc	NBQX	DIV 14–16	↑	↑	[36]
		DIV 17	↑	↑	[51]
		DIV 17	↑	↑	[24]
		DIV 21	N/A	“↑” *	[29]
		DIV 27–40	↑	↑	[49]

Hpc	CNQX	DIV 14	↑	—	[46]
		DIV 21	↑	↑	[46]
		DIV 21–38	↑	↑	[49]

Hpc	Kir2.1 expression	DIV 14-15	—	↑	[45]
		DIV 15–24	↑	N/A	[52]

In vitro (organotypic slice, all from P6-8 cultures)					

Hpc	TTX	DIV 8 (CA3)	↑	↑	[53]
		DIV15 (CA3)	↑	↑	[53]
		DIV 21–25 (MF-CA3)	—	↑	[54]
		DIV 21–25 (CA3-CA3)	—	↓	[54]
		DIV 21–25 (CA3-CA1)	—	—	[54]

Hpc	TTX + APV	DIV 5–7 (CA1)	↑	—	[55]
		DIV 6–8 (CA1)	↑	—	[50]

Ex vivo (acute slice)					

Hpc	TTX ex vivo incubation	P4 (CA3)	↑	↑	[53]
		P8 (CA3)	—	—	[53]
		P21–28 (CA1)	—	—	[35]

Hpc	TTX in vivo implantation	P15 (CA1)	↑	↑	[56]
		P30 (CA1)	—	↑	[56]

Hpc	TTX + APV ex vivo	P21–28 (CA1)	↑	—	[35]

Visual cortex	Intraocular TTX	P21	↑	—	[27]
	Monocular deprivation	P21	↓	↓	[27]
	Binocular deprivation	P23	↑	—	[57, 58]

**Table 2:** Inactivity paradigms: consequences and responses. Inactivity paradigms are grouped by scope: network-wide, cell autonomous, or synapse specific. Each inactivity paradigm is evaluated based on its type: presynaptic (Pre) or postsynaptic (Post) mode of action, and reduction (↓) or elimination (X) of activity.

Paradigm type			Synaptic/cellular consequences	Perceived situation	Cell autonomous response
Network-wide inactivity					

TTX	Pre	↓	*Developing network*: fewer presynaptic inputs; no emergence of AP firing to constrain synapses	Participation in a sparsely connected network	Calibration of synaptic strength to higher level [26, 38, 59] via constitutive insertion of somatically synthesized GluA1/2 AMPARs [34]
			*Established network*: Sudden decrease in output with concurrent decrease in presynaptic inputs	Change in network activity state	Compensation via insertion of somatically synthesized GluA1/2 AMPARs [34] with possible coordination of presynaptic properties (↑ release probability or # synaptic vesicles) or potential ↑ # synaptic sites

APV	Post	↓	Diminished Ca^2+^ influx at synapses	Disrupted synaptic Ca^2+^ homeostasis	Minimal effect at AMPARs [38]

TTX+ APV	Post	↓↓	Sudden decrease in output with concurrent decrease in presynaptic inputs, and diminished synaptic Ca^2+^	Change in network activity state, disrupted synaptic Ca^2+^ homeostasis	Homeostatic compensation via rapid insertion of locally synthesized Ca^2+^ permeable homomeric GluA1 AMPARs [35]

NBQX	Post	X	Sudden decrease in postsynaptic efficacy at an otherwise functional synapse	Disrupted synaptic function and synaptic Ca^2+^ homeostasis	Homeostatic compensation via increase in presynaptic release probability and rapid insertion of locally synthesized Ca^2+^ permeable homomeric GluA1 AMPARs [24, 51]

Cell-autonomous inactivity					

Kir2.1	Post	↓	*Developing network*: less action potential firing than neighbors; less activity-dependent strengthening of synaptic connections	Participation in an “irrelevant” circuit	Inability to compete for synaptic connections in an activity-dependent fashion; lower levels of AMPAR input; lower frequency of inputs (note: this “competition” effect is reversed by global TTX which equalizes activity across the network [45])
			*Established network*: gradual decrease in output without decrease in presynaptic inputs	Decreased postsynaptic efficacy	Homeostatic compensation via increase in presynaptic release probability [45]

Synapse-specific inactivity					

Kir2.1	Pre	↓	Diminished presynaptic input in a normally functioning network	Decreased presynaptic efficacy	Homeostatic compensation via insertion of GluA1 AMPARs [47]

TeTx	Pre	X	Absent presynaptic input in a normally functioning network	Nonfunctional presynaptic terminal	Lack of activity-induced maintenance of GluR1 via diffusional trapping [75]; loss of GluR1 but not GluR2/3 or synaptic proteins [76]

Inactivity paradigms: AP blockade (TTX); NMDAR blockade (APV); AMPAR blockade (NBQX); hyperpolarization (via transfection of Kir2.1 potassium channel); presynaptic release inhibition (via transfection of tetanus toxin, TeTx).

## References

[1] Cannon W (1932). *The Wisdom of the Body*.

[2] Bernard C (1974). *Lectures on the Phenomena Common to Animals and Plants (1878)*.

[3] Burrone J, Murthy VN (2003). Synaptic gain control and homeostasis. *Current Opinion in Neurobiology*.

[4] Nelson SB, Turrigiano GG (2008). Strength through Diversity. *Neuron*.

[5] Pozo K, Goda Y (2010). Unraveling mechanisms of homeostatic synaptic plasticity. *Neuron*.

[6] Zhang W, Linden DJ (2003). The other side of the engram: experience-driven changes in neuronal intrinsic excitability. *Nature Reviews Neuroscience*.

[7] Watt AJ, Desai NS (2010). Homeostatic plasticity and STDP: keeping a neuron’s cool in a fluctuating world. *Frontiers in Synaptic Neuroscience*.

[8] Turrigiano GG (2008). The self-tuning neuron: synaptic scaling of excitatory synapses. *Cell*.

[9] Turrigiano GG, Nelson SB (2000). Hebb and homeostasis in neuronal plasticity. *Current Opinion in Neurobiology*.

[10] Wiener N (1965). *Cybernetics: or, Control and Communication in the Animal and the Machine*.

[11] Francis BA, Wonham WM (1976). The internal model principle of control theory. *Automatica*.

[12] Bakshi U, Bakshi M (2009). *Modern Control Theory*.

[13] Yi TM, Huang Y, Simon MI, Doyle J (2000). Robust perfect adaptation in bacterial chemotaxis through integral feedback control. *Proceedings of the National Academy of Sciences of the United States of America*.

[14] Zhang Q, Pi J, Woods CG, Andersen ME (2010). A systems biology perspective on Nrf2-mediated antioxidant response. *Toxicology and Applied Pharmacology*.

[15] Watson EM, Chappell MJ, Ducrozet F, Poucher SM, Yates JWT (2011). A new general glucose homeostatic model using a proportional-integral-derivative controller. *Computer Methods and Programs in Biomedicine*.

[16] Davis GW (2006). Homeostatic control of neural activity: from phenomenology to molecular design. *Annual Review of Neuroscience*.

[17] O’Leary T, Wyllie DJA (2011). Neuronal homeostasis: time for a change?. *Journal of Physiology*.

[18] Sanes JR, Lichtman JW (1999). Development of the vertebrate neuromuscular junction. *Annual Review of Neuroscience*.

[19] LeMasson G, Marder E, Abbott LF (1993). Activity-dependent regulation of conductances in model neurons. *Science*.

[20] Abbott LF, LeMasson G (1993). Analysis of Neuron Models with Dynamically Regulated Conductances. *Neural Computation*.

[21] Desai NS, Rutherford LC, Turrigiano GG (1999). Plasticity in the intrinsic excitability of cortical pyramidal neurons. *Nature Neuroscience*.

[22] Grubb MS, Burrone J (2010). Activity-dependent relocation of the axon initial segment fine-tunes neuronal excitability. *Nature*.

[23] O’Brien RJ, Kamboj S, Ehlers MD, Rosen KR, Fischbach GD, Huganir RL (1998). Activity-dependent modulation of synaptic AMPA receptor accumulation. *Neuron*.

[24] Thiagarajan TC, Lindskog M, Tsien RW (2005). Adaptation to synaptic inactivity in hippocampal neurons. *Neuron*.

[25] Turrigiano GG, Nelson SB (2004). Homeostatic plasticity in the developing nervous system. *Nature Reviews Neuroscience*.

[26] Wilson NR, Ty MT, Ingber DE, Sur M, Liu G (2007). Synaptic reorganization in scaled networks of controlled size. *Journal of Neuroscience*.

[27] Maffei A, Turrigiano GG (2008). Multiple modes of network homeostasis in visual cortical layer 2/3. *Journal of Neuroscience*.

[28] Prinz AA, Bucher D, Marder E (2004). Similar network activity from disparate circuit parameters. *Nature Neuroscience*.

[29] Murthy VN, Schikorski T, Stevens CF, Zhu Y (2001). Inactivity produces increases in neurotransmitter release and synapse size. *Neuron*.

[30] Malenka RC, Nicoll RA (1997). Silent synapses speak up. *Neuron*.

[31] Kay L, Humphreys L, Eickholt BJ, Burrone J (2011). Neuronal activity drives matching of pre-and postsynaptic function during synapse maturation. *Nature Neuroscience*.

[32] Ripley B, Otto S, Tiglio K, Williams ME, Ghosh A (2011). Regulation of synaptic stability by AMPA receptor reverse signaling. *Proceedings of the National Academy of Sciences of the United States of America*.

[33] Ju W, Morishita W, Tsui J (2004). Activity-dependent regulation of dendritic synthesis and trafficking of AMPA receptors. *Nature Neuroscience*.

[34] Wierenga CJ, Ibata K, Turrigiano GG (2005). Postsynaptic expression of homeostatic plasticity at neocortical synapses. *Journal of Neuroscience*.

[35] Sutton MA, Ito HT, Cressy P, Kempf C, Woo JC, Schuman EM (2006). Miniature Neurotransmission Stabilizes Synaptic Function via Tonic Suppression of Local Dendritic Protein Synthesis. *Cell*.

[36] Groth RD, Lindskog M, Thiagarajan TC, Li L, Tsien RW (2011). *β* Ca^2+^/CaM-dependent kinase type II triggers upregulation of GluA1 to coordinate adaptation to synaptic inactivity in hippocampal neurons. *Proceedings of the National Academy of Sciences of the United States of America*.

[37] Lazarevic V, Schöne C, Heine M, Gundelfinger ED, Fejtova A (2011). Extensive remodeling of the presynaptic cytomatrix upon homeostatic adaptation to network activity silencing. *Journal of Neuroscience*.

[38] Turrigiano GG, Leslie KR, Desai NS, Rutherford LC, Nelson SB (1998). Activity-dependent scaling of quantal amplitude in neocortical neurons. *Nature*.

[39] Gong B, Wang H, Gu S, Heximer SP, Zhuo M (2007). Genetic evidence for the requirement of adenylyl cyclase 1 in synaptic scaling of forebrain cortical neurons. *European Journal of Neuroscience*.

[40] Ibata K, Sun Q, Turrigiano GG (2008). Rapid Synaptic Scaling Induced by Changes in Postsynaptic Firing. *Neuron*.

[41] Wierenga CJ, Walsh MF, Turrigiano GG (2006). Temporal regulation of the expression locus of homeostatic plasticity. *Journal of Neurophysiology*.

[42] Anggono V, Clem RL, Huganir RL (2011). PICK1 loss of function occludes homeostatic synaptic scaling. *Journal of Neuroscience*.

[43] Hu J-H, Park JM, Park S (2010). Homeostatic Scaling Requires Group I mGluR Activation Mediated by Homer1a. *Neuron*.

[44] Han EB, Stevens CF (2009). Development regulates a switch between postand presynaptic strengthening in response to activity deprivation. *Proceedings of the National Academy of Sciences of the United States of America*.

[45] Burrone J, O’Byrne M, Murthy VN (2002). Multiple forms of synaptic plasticity triggered by selective suppression of activity in individual neurons. *Nature*.

[46] Wang H-L, Zhang Z, Hintze M, Chen L (2011). Decrease in calcium concentration triggers neuronal retinoic acid synthesis during homeostatic synaptic plasticity. *Journal of Neuroscience*.

[47] Hou Q, Zhang D, Jarzylo L, Huganir RL, Man HY (2008). Homeostatic regulation of AMPA receptor expression at single hippocampal synapses. *Proceedings of the National Academy of Sciences of the United States of America*.

[48] Sokolova IV, Mody I (2008). Silencing-induced metaplasticity in hippocampal cultured neurons. *Journal of Neurophysiology*.

[49] Jakawich SK, Nasser HB, Strong MJ (2010). Local presynaptic activity gates homeostatic changes in presynaptic function driven by dendritic BDNF synthesis. *Neuron*.

[50] Aoto J, Nam CI, Poon MM, Ting P, Chen L (2008). Synaptic Signaling by All-Trans Retinoic Acid in Homeostatic Synaptic Plasticity. *Neuron*.

[51] Thiagarajan TC, Piedras-Renteria ES, Tsien RW (2002). *α*- and *β*CaMKII: Inverse regulation by neuronal activity and opposing effects on synaptic strength. *Neuron*.

[52] Béïque J-C, Na Y, Kuhl D, Worley PF, Huganir RL (2011). Arc-dependent synapse-specific homeostatic plasticity. *Proceedings of the National Academy of Sciences of the United States of America*.

[53] Huupponen J, Molchanova SM, Taira T, Lauri SE (2007). Susceptibility for homeostatic plasticity is down-regulated in parallel with maturation of the rat hippocampal synaptic circuitry. *Journal of Physiology*.

[54] Kim J, Tsien RW (2008). Synapse-Specific Adaptations to Inactivity in Hippocampal Circuits Achieve Homeostatic Gain Control while Dampening Network Reverberation. *Neuron*.

[55] Soden ME, Chen L (2010). Fragile X protein FMRP is required for homeostatic plasticity and regulation of synaptic strength by retinoic acid. *Journal of Neuroscience*.

[56] Echegoyen J, Neu A, Graber KD, Soltesz I (2007). Homeostatic plasticity studied using in vivo hippocampal activity-blockade: synaptic scaling, intrinsic plasticity and age-dependence.. *PloS one*.

[57] Goel A, Lee HK (2007). Persistence of experience-induced homeostatic synaptic plasticity through adulthood in superficial layers of mouse visual cortex. *Journal of Neuroscience*.

[58] Gao M, Sossa K, Song L (2010). A specific requirement of Arc/Arg3.1 for visual experience-induced homeostatic synaptic plasticity in mouse primary visual cortex. *Journal of Neuroscience*.

[59] Liu G, Tsien RW (1995). Properties of synaptic transmission at single hippocampal synaptic boutons. *Nature*.

[60] Harris KM, Jensen FE, Tsao B (1992). Three-dimensional structure of dendritic spines and synapses in rat hippocampus (CA 1) at postnatal day 15 and adult ages: implications for the maturation of synaptic physiology and long-term potentiation. *Journal of Neuroscience*.

[61] Papa M, Bundman MC, Greenberger V, Segal M (1995). Morphological analysis of dendritic spine development in primary cultures of hippocampal neurons. *Journal of Neuroscience*.

[62] Leslie KR, Nelson SB, Turrigiano GG (2001). Postsynaptic depolarization scales quantal amplitude in cortical pyramidal neurons. *The Journal of neuroscience : the official journal of the Society for Neuroscience*.

[63] Goold CP, Nicoll RA (2010). Single-Cell Optogenetic Excitation Drives Homeostatic Synaptic Depression. *Neuron*.

[64] Deisseroth K, Heist EK, Tsien RW (1998). Translocation of calmodulin to the nucleus supports CREB phosphorylation in hippocampal neurons. *Nature*.

[65] Wheeler DG, Barrett CF, Groth RD, Safa P, Tsien RW (2008). CaMKII locally encodes L-type channel activity to signal to nuclear CREB in excitation-transcription coupling. *Journal of Cell Biology*.

[66] Sutton MA, Schuman EM (2006). Dendritic Protein Synthesis, Synaptic Plasticity, and Memory. *Cell*.

[67] Shepherd JD, Rumbaugh G, Wu J (2006). Arc/Arg3.1 Mediates Homeostatic Synaptic Scaling of AMPA Receptors. *Neuron*.

[68] Pak DTS, Sheng M (2003). Targeted Protein Degradation and Synapse Remodeling by an Inducible Protein Kinase. *Science*.

[69] Seeburg DP, Feliu-Mojer M, Gaiottino J, Pak DTS, Sheng M (2008). Critical Role of CDK5 and Polo-like Kinase 2 in Homeostatic Synaptic Plasticity during Elevated Activity. *Neuron*.

[70] Seeburg DP, Sheng M (2008). Activity-induced polo-like kinase 2 is required for homeostatic plasticity of hippocampal neurons during epileptiform activity. *Journal of Neuroscience*.

[71] Evers DM, Matta JA, Hoe HS (2010). Plk2 attachment to NSF induces homeostatic removal of GluA2 during chronic overexcitation. *Nature Neuroscience*.

[72] Lee K, Lee Y, Rozeboom A (2011). Requirement for Plk2 in orchestrated ras and rap signaling, homeostatic structural plasticity, and memory. *Neuron*.

[73] Branco T, Staras K, Darcy KJ, Goda Y (2008). Local Dendritic Activity Sets Release Probability at Hippocampal Synapses. *Neuron*.

[74] Zhao C, Dreosti E, Lagnado L (2011). Homeostatic synaptic plasticity through changes in presynaptic calcium influx. *Journal of Neuroscience*.

[75] Ehlers MD, Heine M, Groc L, Lee MC, Choquet D (2007). Diffusional Trapping of GluR1 AMPA Receptors by Input-Specific Synaptic Activity. *Neuron*.

[76] Harms KJ, Tovar KR, Craig AM (2005). Synapse-specific regulation of AMPA receptor subunit composition by activity. *Journal of Neuroscience*.

[77] Gainey MA, Hurvitz-Wolff JR, Lambo ME, Turrigiano GG (2009). Synaptic scaling requires the GluR2 subunit of the AMPA receptor. *Journal of Neuroscience*.

[78] Kim CH, Lisman JE (2001). A labile component of AMPA receptor-mediated synaptic transmission is dependent on microtubule motors, actin, and N-ethylmaleimide-sensitive factor. *Journal of Neuroscience*.

[79] Lee MC, Yasuda R, Ehlers MD (2010). Metaplasticity at Single Glutamatergic Synapses. *Neuron*.

[80] Sutton MA, Wall NR, Aakalu GN, Schuman EM (2004). Regulation of dendritic protein synthesis by miniature synaptic events. *Science*.

[81] Pannasch U, Vargova L, Reingruber J (2011). Astroglial networks scale synaptic activity and plasticity. *Proceedings of the National Academy of Sciences of the United States of America*.

[82] Stellwagen D, Malenka RC (2006). Synaptic scaling mediated by glial TNF-*α*. *Nature*.

